# Tolerance and safety evaluation of L-glutamic acid, N,N-diacetic acid as a feed additive in broiler diets

**DOI:** 10.1016/j.psj.2021.101623

**Published:** 2021-11-29

**Authors:** Gavin Boerboom, Javier Martín-Tereso, Teun Veldkamp, Jan van Harn, Paul Bikker, Ronald Busink

**Affiliations:** ⁎ Animal Nutrition Group, Wageningen University & Research, Wageningen 6708 WD, the Netherlands; † Trouw Nutrition R&D, Amersfoort 3811 MH, the Netherlands; ‡ Wageningen Livestock Research, Wageningen University and Research, 6708 WD, Wageningen, the Netherlands

**Keywords:** GLDA, trace mineral, broiler, tolerance, feed additive

## Abstract

The novel chelator, L-glutamic acid, N,N-diacetic acid (**GLDA**) can be used as a dietary ingredient to safely reduce Zn supplementation in complete feed, without compromising the Zn status of farm animals. The objective of this study was to study dietary tolerance, bioaccumulation, and evaluate the safety of GLDA when supplemented in broiler diets at 0, 100, 300, 1000, 3,000, and 10,000 mg/kg. A total of 480 one-day-old Ross 308 male broilers were randomly allocated to 48 pens and fed one of the 6 experimental diets. Production performance was used to assess tolerance to the additive. At trial end, toxicity was evaluated using hematology, plasma biochemistry (n = 144) and gross necropsy (n = 48). Residue levels of GLDA were assessed in liver, kidney and breast tissue of birds used for necropsy. Performance showed an increase (*P <* 0.05) in body weight for GLDA inclusion at 300 mg/kg. A decrease on the measured performance parameters was found for the 10,000 mg/kg GLDA inclusion level (*P <* 0.05). The additive was added as a tetra-sodium salt, leading to sodium levels being 2.5 times higher in the latter treatment compared to the control diet which may have led to impaired intestinal barrier function. Mortality was not different between treatments. Residue levels for GLDA at the highest inclusion indicate that 0.0005% of total GLDA consumption is accumulated in breast tissue. Higher values of GLDA were found in kidney and liver at the highest inclusion level, potentially confirming that the small fraction of GLDA absorbed was readily excreted by the animal. At 100 and 300 mg/kg GLDA inclusion there were negligible amounts of GLDA present in all tissues measured. The present experiment demonstrated a high dietary tolerance to GLDA in broilers and indicated that GLDA does not pose a significant risk to food safety when supplemented below 3,000 mg/kg.

## INTRODUCTION

Trace minerals, such as zinc (**Zn**) and copper (**Cu**), are vital nutrients to ensure human and animal health (Richards et al., 2010; Rink, 2011). Zinc for example serves as a cofactor for over 300 enzymes and 2,000 transcription factors. Minerals can be absorbed from any segment of the gastrointestinal-tract but are mainly absorbed in the small intestines (Rink, 2011; Svihus, 2014; Yu et al., 2017). Many factors may influence the absorption of minerals, such as dietary antagonists (phytic acid), dietary levels of other minerals (Cu vs Zn), interactions between different minerals within the gastrointestinal-tract and even the interaction of minerals with the microbiota (Humer et al., 2015; Brugger and Windisch, 2017; Brugger and Windisch, 2019). The chemical form in which Zn is supplemented to diets also directly affects its bioavailability in a complete diet. In animal systems, Zn is often supplemented above the requirements (120 ppm vs 40 ppm) to compensate for the uncertainty in bioavailability defined by the factors described above. This practice results in a low relative efficiency of Zn utilization, while producing a higher Zn excretion into animal manures (Weigand and Kirchgessner, 1980). This higher excretion of Zn and other excessively supplemented trace minerals contributes to environmental pollution due to animal production (Brugger and Windisch, 2015; Brugger and Windisch, 2019).

Single strong chelating agents in diets are able to increase mineral bioavailability. Chelating agents comprise of molecules with a high affinity to bind trace elements and keep them in solution. During digestion, the formation of this stable complex in the upper gastrointestinal tract minimizes the formation of insoluble complexes, resulting in a preservation of nutritional bioavailability (Krezel and Maret, 2016). Chelators such as ethylenediaminetetraacetic acid (**EDTA**) have been used in both human and animal diets to increase bioavailability of minerals (MacPhail et al., 1994; Heimbach et al., 2000; Hurrell et al., 2000). In humans, iron EDTA has shown to have a beneficial effect on iron status (Davidsson et al., 1994; Heimbach et al., 2000). A novel chelator, L-glutamic acid, N,N-diacetic acid (**GLDA**) has shown to increase the nutritional availability of Zn in broilers. In this way, it can be used as a dietary ingredient to safely reduce Zn supplementation in complete feed without compromising the Zn status of the animal, thus contributing to the reduction of environmental trace element pollution of animal production (Kołodyńska, 2011; Wu et al., 2015; Boerboom et al., 2020; Boerboom et al., 2021). In contrast with other chelators such as EDTA, GLDA is readily biodegradable in the environment presenting a low persistence in soils and surface waters. Adverse effects were observed for dietary EDTA inclusion at higher concentrations, including induction of oxidative stress, tissue injury and disruption of tight junction and membrane integrity (Prachayasittikul et al., 2007). It is understood that the high chelation strength of EDTA affects the metal ions in the outer membrane, resulting in lipopolysaccharide and protein dissociation. Whether GLDA, being a strong chelator as well, would cause such adverse effects and at which dietary concentration remains unclear. The current experiment was conducted aims to evaluate tolerance and safety of the use of GLDA by inclusion in broiler diets at incremental levels up to a dose of 100-fold of the lowest recommended dosed as used in the previous study (Boerboom et al., 2021). In addition, the bioaccumulation of GLDA in different edible tissues was determined to address potential effects on food safety. The hypothesis was that dietary inclusion of GLDA does not cause negative effects on broiler performance or does not increase bioaccumulation in broiler tissues within the range of inclusion levels studied.

## METHODS

The implementation of the trial and experimental design were defined in accordance with the technical guidance, as stipulated by European Feed Safety Association Panel on Additives and Products or Substances used in Animal Feed (Additives and Feed, 2011). The experiment was performed in compliance with the Dutch legislation for animal experiments, and the protocol was approved by the Committee for Animal Experiments of Wageningen University and Research, the Netherlands.

### Animals

This study was conducted in the facilities of Wageningen Bioveterinary Research, Building 161, Lelystad, the Netherlands, using 480 one-day-old Ross 308 male broilers. A total of 48 pens (0.75m^2^) bedded with wood shavings were used in the experiment. Each pen contained one feeder bin and a drinking line with 2 drink cups providing water and feed ad libitum. Each pen had 10 male broilers from d 0 to 35. Six dietary treatments were randomly allocated to the pens within blocks to include environmental factors within the room into a statistical block factor. At d 10, 3 birds from each pen were randomly selected to be used for representative sampling of blood and tissues at the end of the study.

### Diets

Dietary treatments consisted of 6 incremental levels of GLDA [0, 100, 300, 1,000, 3,000, 10,000 mg/kg of GLDA, i.e. 0, 333, 999, 3,330, 9,990 and 33,300 mg/kg of GLDA-silica premix (30% GLDA)] (Trouw Nutrition, Amersfoort, the Netherlands) and a fixed dose of supplemental Zn (100 mg/kg of Zn from ZnSO_4_.H_2_O). The experimental diets were fed from d 0 to 35 of age. A corn-soybean meal based diet was formulated to fulfill all standard nutritional requirements (NRC, 1994) (Table 1). One basal meal was prepared and subdivided for the production of the treatment specific diet. All diets were supplemented with 100 mg/kg Zn (from ZnSO_4_.H_2_O), 10 mg/kg of Cu (from CuSO_4_.H_2_O), 100 mg/kg of manganese (from MnO), and 120 mg/kg of iron (from FeSO_4_.H_2_O) by the use of a premix. The diet supply was subdivided in 2 feeding phases, the starter from d 0 to 10 and the grower from d 11 to d 35 of age. All experimental diets were formulated and produced by Research Diet Services B.V., Wijk bij Duurstede, the Netherlands.

**Table 1:** Calculated ingredient composition of the basal mixture for the experimental diets supplied to broilers in the starter phase from d 0 to 10 d of age and the grower phase from d 11 to 35 d of age.

Ingredients (g/kg)	Starter	Grower
Corn	578.9	604.7
Soybean meal	345.0	310.0
Soybean Oil	36.5	52.5
L-Lysine	0.7	1.4
DL-Methionine	1.9	2.0
L-Threonine	0.0	0.3
Limestone	14.0	11.0
Monocalcium phosphate	13.0	8.5
Salt	2.5	2.5
NaHCO_3_	2.5	2.1
Premixture^1^	5.0	5.0
Total	1,000	1,000
Metabolizable energy broilers (MJ/kg)	11.91	12.56
Crude protein	217	203
Fat	61	78
Crude fiber	25	24
Starch	382	396
Calcium	9.0	7.0
Phosphorus, total	6.3	5.2
Phosphorus, digestible	3.6	2.7
Magnesium	1.7	1.5
Potassium	9.7	9.0
Sodium	1.7	1.6
Chlorine	2.0	2.2
Digestible lysine	10.5	10.2
Digestible methionine	4.8	4.7
Digestible methionine+cysteine	7.7	7.4
Digestible threonine	6.8	6.6
Digestible trypsin	2.2	2.0
Arginine	13.0	12.0

1 Supplemented vitamin and mineral levels per kg feed based on a 0.5% inclusion level of premixture: 12,000 IU vitamin A, 2,400 IU vitamin D3, 50 mg vitamin E, 1.5 mg vitamin K3, 2 mg vitamin B1, 7.5 mg vitamin B2, 35 mg niacin amide (vitamin B3), 12 mg d-pantothenic acid (vitamin B5), 3.5 mg vitamin B6, 0.2 mg biotin, 20 μg vitamin B12, 1 mg folic acid, 460 mg choline chloride, 0.4 mg Co (as CoSO_4_.7H_2_O), 0.8 mg I (as KI), 0.15 mg Se (as Na_2_SeO_3_) and 125 mg antioxidant. 120 mg Fe (as FeSO_4_.H_2_O), 10 mg Cu (as CuSO_4_.5H_2_O), 100 mg Zn (as ZnSO_4_.H_2_O), and 100 mg Mn (as MnO) were added.

### Diet Analysis

Samples of the basal meals were analyzed for moisture (EC No 152/2009), ash (EC No 152/2009), protein (ISO 5983-2-2009), crude fat (EC No 152/2009 method B), crude fiber (KWAWRC equivalent to NEN-EN ISO 6865), and trace minerals (Zn, Cu, Mn, Fe) (AAS in following NEN-EN ISO 6869) (Pre Mervo, Utrecht, the Netherlands). The experimental diets were analyzed for moisture, Zn and GLDA. L-glutamic acid, N,N-diacetic acid content was measured in duplicate by using liquid chromatography-mass spectrometry (**LC-MS**) (Masterlab B.V., Boxmeer, the Netherlands). L-glutamic acid, N,N-diacetic acid was quantitatively water extracted from ground feed samples. The extracted GLDA and other extracted components from the experimental diets were separated applying a reversed phase liquid chromatography, using an Alltima C18 AQ 3 μm column as stationary phase and 0.2 % tri-fluoroacetic acid in water as mobile phase. The GLDA molecule was detected at m/z 264.070 using a Triple Quadrupole LC-MS mass spectrometer. L-glutamic acid, N,N-diacetic acid levels in feed samples were calculated using a GLDA standard curve based on a calibrated GLDA standard.

### Measurements

Observations included performance parameters, body weight (**BW**), feed intake (**FI**), body weight gain (**BWG**), daily weight gain (**DWG**), and feed conversion ratio (**FCR**), at 0, 10 and 35 d of age. Mortality was registered and the cause of death was determined by necropsy. At the end of the study, at d 35 of age, 3 marked birds were used to collect 5 blood samples from each bird, taken from the wing veins: 2x serum tube, 1 EDTA tube, 1 NaF tube and 1x Zn-free tube. The Zn-free tube was centrifuged immediately after sampling and aliquoted in coded cryotubes (Centrifuge Centra CL3r, serial number 37560759, serum tubes 3,000 rpm/1,800 rcf 8 min, 18°C, heparin blood tubes 3,000 rpm/1,800 rcf 4 min 4°C). The cryotubes were stored frozen at -80°C until shipment. Serum samples were sent frozen in a cool box (with dry ice) to the Glasgow Royal Infirmary, Glasgow, United Kingdom, and analyzed for Zn, Cu, Mn, and Fe content. Samples were centrifuged because precipitate was visually evident in several of the samples. Subsequently, samples were diluted 1:10 with 0.1% EDTA, 0.1% Triton X, 2% butan-1-ol, 1% ammonia and 50 µg/L germanium, and scandium as internal standards. Analysis was conducted by inductively couple plasma mass spectrometry using a helium reaction cell. The remaining blood samples were analyzed by the Animal Health Service (GD), Deventer, the Netherlands, for general hematology assessment and routine blood chemistry including: white blood cells, red blood cells, hemoglobin, hematocrit, mean corpuscular volume, mean corpuscular hemoglobin, differential leucocyte count (hematology analyzer), albumin, gamma-glutamyl transferase, aspartate aminotransferase, alanine aminotransferase, urea, total protein, glucose, alkaline phosphatase, lactate dehydrogenase, albumin/globulin, creatinine, calcium, phosphorus, total bilirubin (Analyzer−ultraviolet-visible spectroscopy), chlorine, sodium, and potassium (ISE) (Additives and Feed, 2011). On d 35, one bird from each pen was randomly selected for gross necropsy. Birds were humanely killed by T61 (an aqueous solution containing (in mg per mL) embutramide, 200 mg/mL; mebezoniumiodide, 50 mg/mL; tetracainehydrochloride, 5 mg/mL) via wing vein injection. General necropsy reports focused primarily on kidney and liver abnormalities. Samples of breast muscle, liver, and kidney were taken for GLDA content analysis (from one bird from each pen receiving 100, 300 and 10,000 mg/kg GLDA). These samples were frozen at -18ᵒC until further analysis. The frozen organ tissue was cut into 3 equal pieces using a surgical blade knife. From each 3 sub-samples approximately 0.5 to 1.0 g tissue was dissected and cut into smaller pieces. 0.5 g of tissue (weight was recorded to the nearest 0.01 g) was then transferred into a 2 mL volume micro-centrifuge tube containing ± 0.5 g ceramic beads (1.4 mm) and 1.0 mL of dimethyl sulfoxide. The mixture was vigorously agitated using a Magna Lyser for 3 × 20 s at 6,000 rpm velocity. After cell disruption the mixture was centrifuged for 10 min at 17,000 rpm/26,810 rcf. The supernatants of the 3 subsamples were collected and pooled to obtain one cell-free extract per tissue sample. For analysis of GLDA in a cell-free extract, GLDA was separated applying reversed phase liquid chromatography, using a Synnergy Polar C18 4 µm column as stationary phase and 0.2 % trifluoroacetic acid in water as mobile phase. The GLDA molecules were detected at m/z 264.07 using a TSQ Endura Triple Quadrupole Mass Spectrometer with turbo pump and rotary prevacuum pump, fitted with a Heated Electro Spray Ionization probe. GLDA levels were calculated against standard curves of GLDA, prepared from calibrated GLDA standards. The limit of detection and limit of quantification for GLDA were determined at 0.5 and 1.0 µg/kg respectively. The developed LC-MS method was therefore considered sufficiently sensitive to measure relevant levels of GLDA.

### Statistical Analysis

Data were analyzed using SAS Studio (SAS institute Inc., Cary, NC). Outliers were checked using the influence statement within the procedures applied. Performance data, serum mineral concentration, GLDA residues in body tissues and blood characteristics were analyzed using the MIXED procedure. L-glutamic acid, N,N-diacetic acid dose was the main effect with block as random effect and time (if applicable) as a repeated effect. Pen was considered the experimental unit.

Y=μ+GLDA+Block+Error

Y = Response parameter
µ = General mean
GLDA = effect of GLDA
Block = Effect of block
Error = Error term

Main effects with *P <* 0.05 were considered to be statistically significant and a *P*-value between 0.05 and 0.10 was considered a trend. Significantly different means were identified between the different levels of GLDA inclusion with a Tukey test (*P <* 0.05). Linear and quadratic effects of GLDA were also determined using the MIXED procedure.

## RESULTS

GLDA recovery in the starter diets for 100, 300, 1,000, 3,000, and 10,000 was slightly below dosing, representing 87, 94, 95, 92, and 89% of the dose respectively (Table 2). Similarly, GLDA recovery in the grower diets for 100, 300, 1,000, 3,000, and 10,000 was 91, 95, 91, 92, and 98% respectively. The results of the proximate analysis of the starter and grower diet met the calculated contents (90–110% of expected values). Analyzed Zn content was lower than calculated, but in a consistent way across treatments (Table 2).

**Table 2:** Analyzed Zn and L-glutamic acid, N,N-diacetic acid (GLDA) content in experimental diets.

Expected GLDA	Starter		Grower	
	Analyzed Zn	Analyzed GLDA	Analyzed Zn	Analyzed GLDA
0	96	0	85	0
100	90	87	87	91
300	92	283	88	286
1,000	91	948	86	905
3,000	90	2,769	82	2,752
10,000	97	8,904	88	9,792

Dietary GLDA inclusion body weight, feed efficiency, feed intake and daily weight gain in the total period and in the grower period (*P <* 0.05) (Table 3). Quadratic responses were observed for the grower and total period for all parameters except feed intake (*P <* 0.05). GLDA inclusion at 1,000 and 3,000 mg/kg improved FCR over the entire period compared to the control, while GLDA at 1,000 mg/kg improved FCR in the starter phase as well (*P <* 0.05). Inclusion of GLDA at the highest dose (10,000 mg/kg) negatively affected all performance parameters apart from FCR in the starter phase (*P <* 0.05).

**Table 3:** Effect of dietary L-glutamic acid, N,N-diacetic acid (GLDA) inclusion from d 0 to 35 on growth performance of broilers (LSmeans).

GLDA, mg/kg	0	100	300	1,000	3,000	10,000	SEM	GLDA	Linear	Quadratic
BW d 0, g	44.1	44.3	44.0	44.2	44.2	44.1	0.04	0.36	0.26	0.25
BW d 10, g	291	293	289	290	287	277	1.7	0.11	0.47	0.95
BW d 35, g	2338	2348	2439*	2377	2374	2044*	23	<.0001	0.32	0.01
FCR 0–10 d	1.13	1.11	1.10	1.10	1.11	1.11	0.004	0.27	0.34	0.33
FCR 10–35 d	1.48	1.49	1.47	1.46	1.45*	1.53*	0.005	<.0001	<0.01	<.0001
FCR 0–35 d	1.44	1.45	1.43	1.42*	1.41*	1.48*	0.004	<.0001	<0.01	<.0001
FI 0–10 d, g	278	275	269	270	268	259*	2	0.09	0.23	0.53
FI 10–35 d, g	3026	3064	3151†	3046	3021	2702*	27	<.0001	0.74	0.23
FI 0–35 d, g	3304	3339	3420†	3316	3289	2961*	28	<.0001	0.66	0.27
DWG 0–10 d, g	24.6	24.8	24.5	24.6	24.2	23.3	0.17	0.11	0.46	0.93
DWG 10–35 d, g	81.9	82.2	86.0*	83.5	83.5	70.7*	0.88	<.0001	0.26	0.01
DWG 0–35 d, g	65.5	65.8	68.4*	66.7	66.6	57.1*	0.66	<.0001	0.32	0.01

⁎ Indicates significant difference compared to the control (*P <* 0.05).

† Indicates trend compared to control (*P* : 0.05–0.10). Abbreviations: BW, bodyweight; DWG, daily weight gain; FCR, feed conversion rate; FI, feed intake.

Serum mineral levels showed that GLDA inclusion in feed resulted in an effect on Zn levels only (*P <* 0.05) (Table 4). Inclusion of GLDA at 10,000 mg/kg tended to increase serum Cu (*P <* 0.10), without a main effect of GLDA or linear/quadratic effects. Serum Fe levels tended to respond quadratically (*P <* 0.10) to GLDA inclusion, showing a numerical decrease in the intermediate level.

**Table 4:** Effect of dietary L-glutamic acid, N,N-diacetic acid (GLDA) inclusion from d 0 to 35 on serum mineral concentration of broilers (LSmeans).

GLDA, mg/kg	0	100	300	1,000	3,000	10,000	SEM	GLDA	Linear	Quadratic
Serum Zn, µg/L	1,391	1,511	1,512	1,565	1,620	1,926*	36	<0.0001	0.09	0.63
Serum Cu, µg/L	96	100	106	104	87	135†	6.1	0.31	0.38	0.19
Serum Fe, mg/L	1.31	1.38	1.29	1.21	1.24	1.37	0.03	0.29	0.11	0.08
Serum Mn, µg/L	16.0	14.7	14.0	11.8	18.0	15.0	1.3	0.85	0.54	0.56

⁎ Indicates significant difference compared to the control (*P <* 0.05).

† Indicates trend compared to control (*P*: 0.05-0.10).

Hematology assessment indicated an effect of GLDA inclusion on erythrocytes, hemoglobin, mean corpuscular hemoglobin, hematocrit, bilirubin, alkaline phosphatase, γ-glutamyl transferase, total protein, albumin and albumin/globulin ratio (*P <* 0.05) (Table 5). The response was quadratic for erythrocytes, mean corpuscular hemoglobin, bilirubin, lymphocytes, sodium, alanine aminotransferase, lactate dehydrogenase, total protein, and glucose. Sodium and chlorine levels were elevated in broilers receiving the experimental diets containing GLDA at 10,000 mg/kg compared to broilers receiving the control diets. Alkaline phosphatase activity was reduced in broilers receiving the experimental diets containing GLDA at levels above 300 mg/kg compared to the broilers receiving the control diet.

**Table 5:** Effect of dietary L-glutamic acid, N,N-diacetic acid (GLDA) inclusion from d 0 to 35 on general hematology assessment and blood chemistry of broilers (LSmeans).

GLDA, mg/kg	Reference value^1^	0	100	300	1,000	3,000	10,000	SEM	GLDA	Linear	Quadratic
Hematology											
Erythrocytes count, 10^12/L	2.3-3.0	2.5	2.6	2.5	2.4	2.5	3.0*	0.04	<0.001	0.38	0.05
Hemoglobin, mmol/L	2.0-5.7	4.5	4.7	4.5	4.5	4.7	5.2*	0.06	0.001	0.50	0.71
Mean corpuscular hemoglobin, fmol	1.7-3.4	1.8	1.8	1.8	1.8	1.9*	1.7	0.01	0.006	0.001	0.0003
Mean corpuscular volume, fL	120-130	123	125	125	125	125	125	0.3	0.17	0.40	0.40
Hematocrit, L/L	0.31-0.55	0.31	0.32	0.31	0.31	0.31	0.37*	0.005	0.001	0.44	0.07
Bilirubin, μmol/L	4.5-9.2	2.3	2.5†	2.2	2.2	2.1	2.3	0.03	0.02	0.02	0.02
Leucocyte count, 10^9/L	20.0-24.0	26.7	24.5	27.4	26.2	23.4	30.6	0.76	0.11	0.15	0.06
Heterophil granulocytes, 10^9/L	6.3-6.5	11.9	11.1	11.8	12.3	10.6	14.7*	0.42	0.10	0.33	0.12
Lymphocytes, 10^9/L	13.9-16.1	13.9	13.0	14.1	13.2	11.5	15.5	0.51	0.31	0.09	0.05
Monocytes, 10^9/L	1.2-1.4	0.88	0.83	1.22	1.18	1.30	1.87*	0.12	0.17	0.35	0.71
Electrolytes											
Chlorine, mmol/L	100-112	111	111	110	110	110	113*	0.33	0.07	0.63	0.24
Calcium, mmol/L	2.0-4.5	2.6	2.6	2.5	2.5	2.5	2.6	0.02	0.69	0.36	0.32
Phosphate, mmol/L	0.65-1.45	3.2	3.0	3.1	2.9†	3.0	3.2	0.04	0.35	0.32	0.20
Potassium, mmol/L	3-5	11.4	10.4	11.6	9.9†	10.7	11.0	0.26	0.39	0.40	0.38
Sodium, mmol/L	140-160	149	151	149	149	150	156*	0.5	<.0001	0.40	0.01
Enzymes											
ALP, IU/L	1884-5822	12700	10776	9669*	7993*	9566*	6724*	486	0.004	0.16	0.43
ALT, IU/L	6-7	5.8	5.6	5.6	6.0	6.4	5.7	0.13	0.51	0.05	0.05
AST, IU/L	179-330	418	387	431	391	456	371	15.5	0.60	0.29	0.21
GGT, IU,L	16-46	20.4	21.2	18.4	19.5	17.8†	23.6*	0.47	0.001	0.02	0.003
LDH, IU/L	378-416	4575	2892	3333	2806	7761†	3614	530	0.05	0.01	0.01
Metabolites											
Total protein, g/L	27-36	33.3	35.8†	33.6	32.8	33.0	38.7*	0.46	<.0001	0.08	0.007
Albumin, g/L	14-20	15.3	16.4*	15.5	15.2	15.7	17.9*	0.21	<.0001	0.54	0.10
Albumin/Globulin ratio	0.78-0.92	0.85	0.85	0.87	0.87	0.91*	0.87	0.005	0.002	<.0001	<.0001
Urea (mmol/L)	0.3-2.5	<2	<2	<2	<2	<2	<2	n.a.	n.a.	n.a.	n.a.
Glucose, mmol/L	11.1-25.0	29.4	29.8	28.8	29.0	32.4	25.8	0.72	0.22	0.11	0.05
Creatinine, μmol/L	22-57	23.8	17.5†	17.4†	20.1	25.3	21.9	1.19	0.15	0.11	0.14

Abbreviations: ALP, alkaline phosphatase; ALT, alanine aminotransferase; AST, aspartate aminotransferase; GGT, γ-glutamyl transferase; LDH, lactate dehydrogenase; n.a., not available.

⁎ Indicates significance compared to the control (*P <* 0.05).

† Indicates trend compared to control (*P*: 0.05–0.10)^1^ (Al-Hussary and Kudair, 2010; Andretta et al., 2012; Igene et al., 2012; Kalmar et al., 2012; Piotrowska et al., 2011; Rezende et al., 2017; Silva et al., 2007; Talebi et al., 2005).

No differences were observed in total mortality while the cause of death showed no specific pattern related to the GLDA dosages administered (Table 6). The results of necropsy indicated a decrease in live weight of the necropsied broilers receiving GLDA at a concentration of 10,000 mg/kg compared to birds receiving the control diets (Table 7). Kidney weight, expressed as a percentage of liveweight, was higher in birds receiving GLDA at 10,000 mg/kg compared to the control. An increase in GLDA residues was observed in tissues of birds receiving the experimental diets containing 10,000 mg/kg of GLDA compared to the tissues of birds receiving 100 and 300 mg/kg of GLDA (Table 8). GLDA residue levels observed in the tissue of birds receiving 100 and 300 mg/kg of GLDA were low (<0.14 mg/kg).

**Table 6:** Causes of mortality in broilers fed dietary L-glutamic acid, N,N-diacetic acid (GLDA) from d 0 to 35.

GLDA, mg/kg	0	100	300	1,000	3,000	10,000
Yolk sac inflammation	-	-	1	1	-	-
Broken femur head	-	-	1	1	-	-
Enlarged liver and liver rupture	-	-	1	-	-	-
Proventricular dilatation, intestinal disorder	-	1	1	-	1	-
Intestinal disorder	4	1	1	1	2	-
Polyserositis	3	2	-	-	-	1
Sudden death syndrome	1	-	-	-	-	-
Pericarditis	-	-	-	-	1	1
No abnormalities detected	-	-	2	2	1	2
Autolyse, no diagnosis could be determined	-	-	-	1	-	-
Total	8	4	7	6	5	4

**Table 7:** Effect of dietary L-glutamic acid, N,N-diacetic acid (GLDA) inclusion from d 0 to 35 on the organ weights of broilers (LSmeans).

GLDA, mg/kg	0	100	300	1,000	3,000	10,000	SEM	GLDA	Linear	Quadratic
Body weight d 35, g	2,214	2,316	2407†	2,366	2,272	1,988*	33.4	0.002	0.82	0.27
Liver, g	48.9	51.9	49.8	52.3	48.5	44.9	0.87	0.15	0.73	0.86
Liver, % of body weight	2.21	2.24	2.07	2.23	2.13	2.28	0.04	0.64	0.58	0.46
Kidney, g	15.2	15.0	16.5	16.7†	16.2	15.6	0.26	0.3	0.21	0.19
Kidney, % of body weight	0.68	0.65	0.69	0.70	0.70	0.79*	0.01	0.005	0.17	0.59

⁎ Indicates significance compared to the control (*P <* 0.05).

† Indicates trend compared to control (*P*:0.05–0.10).

**Table 8:** Effect of dietary L-glutamic acid, N,N-diacetic acid (GLDA) inclusion from d 0 to 35 on GLDA residues in broiler tissues at 35 d of age of broilers (LSmeans).

Tissue	GLDA inclusion (mg/kg)	Average GLDA residue level (mg/kg)
Breast meat	100	0.008^a^ ± 0.003
	300	0.021^a^ ± 0.008
	10,000	0.378^b^ ± 0.102
Liver	100	0.010^a^ ± 0.002
	300	0.018^a^ ± 0.004
	10,000	1.35^b^ ± 0.12
Kidney	100	0.100^a^ ± 0.019
	300	0.131^a^ ± 0.018
	10,000	3.99^b^ ± 0.37

Superscripts indicate significant differences (*P <* 0.05). Averages are given as means + SE.

## DISCUSSION

Analyzed Zn levels were approximately around 70% of expected values. Differences in Zn levels among diets were small, which indicates that the Zn levels within the premix likely were lower than intended. Since the deviation in Zn content was consistent across all diets, it does not impair the contrast of the hypothesis.

Dietary supplementation of GLDA up to a level of 3,000 mg/kg showed no negative effects on any of the performance parameters. Growth performance of the birds in this study was above the Ross 308 guidelines (Aviagen, 2014). Dietary supplementation of 300 mg/kg of GLDA improved final body weight (D 35, *P <* 0.05). This was unexpected as trace mineral levels in the feed were higher than the levels required for adequate growth, even though they were lower than calculated (Mohanna and Nys, 1999; Schlegel et al., 2010). It indicates that GLDA inclusion in a diet may improve performance of broilers even when minerals supply is assumed to be adequate. In a similar way, the current experiment showed an improved FCR with GLDA inclusion of 1,000 and 3,000 mg/kg (*P <* 0.05).

On the other hand, dietary supplementation of 10,000 mg/kg of GLDA reduced performance in the grower period as compared to the group receiving no GLDA and below the Ross 308 performance objectives (BW, DWG, and FCR) (Table 3) (Aviagen, 2014). This may unequivocally be the result of intolerance to dietary GLDA supply. The product was included in the diet as a tetra-sodium salt and the subtotal mass percentage of sodium within GLDA-Na_4_ is 26.19%. Feeding the GLDA molecule at 10,000 mg/kg led to an additional 2,619 mg/kg of sodium in this dietary treatment, that is, 2.5 times higher level compared to the control. This resulted in higher levels of sodium in blood at the highest inclusion level compared to the control (Table 5).

In addition, a higher amount of Zn in serum was detected in birds receiving the 10,000 mg/kg of GLDA. The higher serum Zn concentration could be indicative of impaired intestinal barrier function, potentially resulting from the elevated dietary sodium (Baloš et al., 2016; Tanaka and Itoh, 2019). As an alternative explanation, increased gut permeability could be explained by the chelating agent itself. Strong chelators, such as EDTA, have been described to destabilize membranes when present at high concentrations (Banin et al., 2006; Prachayasittikul et al., 2007). This seems to happen by intercalation between EDTA and the phospholipid molecules through salt bridge formation, by reaction with the polar head of phospholipids. This can mechanically stress the bilayer phospholipid organization leading to membrane disruption and leakage (Prachayasittikul et al., 2007). Considering the much higher chelation strength toward Zn of EDTA compared to GLDA (log K_16.5_ vs log K_10_) it is more plausible that the increased permeability results from increased osmolality due to the higher sodium content.

Mortality of broilers was not increased in the treatment group receiving the experimental diets containing 10,000 mg/kg GLDA, indicating that while this level was inadequate for health or performance; it still was a tolerable dose.

Inclusion of GLDA did not affect serum levels of Mn and Fe, in accordance with previous findings (Boerboom et al., 2020; Boerboom et al., 2021). Copper levels when GLDA was fed at 10,000 mg/kg showed numerically higher values compared to the control, which can be explained by the gut integrity effects described above (Kołodyńska, 2013). The absence of effects at serum level for these minerals is in line with expectations, because all diets contained nutritionally adequate levels for all minerals and as such, chelation is not expected to result in differences in absorption despite of any difference in availability due to down-regulation of absorption (Mondal et al., 2010).

Surprisingly, dietary inclusion of GLDA at 3,000 mg/kg increased the albumin to globulin ratio in comparison to the control broilers, while still being within the reference values (Rezende et al., 2017). Total protein and albumin itself were not affected by this treatment and therefore it can be concluded that the difference in albumin to globulin ratio was not the result of increased inflammatory processes (Rezende et al., 2017). Alkaline phosphatase activity was lower when GLDA was included at all levels higher than 300 mg/kg. This shows the inconsistency that the use of alkaline phosphatase as a marker for Zn status has. (Al-Daraji and Amen, 2011; Amen and Al-Daraji, 2011). Across treatments, potassium levels were higher than reference values and this might indicate pseudo hyperkalemia. This is typically caused by hemolysis during venepuncture and it is a laboratory artifact rather than a biological abnormality (Sevastos et al., 2006). Heterophil granulocyte levels were elevated compared to reference values in all broilers and elevated when feeding GLDA at 10,000 mg/kg (Andretta et al., 2012). Heterophil granulocytes are part of the innate immune system and are the first line of defense against pathogenic infections, which is in line with the hypothesis of increased gut permeability (Bojesen et al., 2004). The mortality numbers and performance results however do not show any indication of decreased performance or disease pressure.

Necropsy detected no hepatic changes. However, kidney weight, expressed as a percentage of live weight, showed an increase in birds receiving 10,000 mg/kg of GLDA (*P <* 0.05). This effect could also result from the levels of sodium in this diet due to GLDA being supplied as a tetrasodium salt. An increase in kidney size has been reported in literature in association to excessive dietary levels of sodium (Mushtaq et al., 2014).

As a reference, the absorption of EDTA in human subjects is described to be around 5% and the pharmacokinetics is similar in experimental animals as compared to humans (Heimbach et al., 2000). Studies in rats have shown that most of the ingested EDTA was not absorbed and the fraction that was absorbed was to a large extent excreted through the urine (Foreman et al., 1953; Heimbach et al., 2000). The total lifetime consumption of GLDA in the current study, when included at 100, 300 or 10,000 mg/kg, would be 352, 1056, and 35200 mg of GLDA, respectively. The fraction of GLDA found in breast tissue is estimated to be 0.01% of total GLDA assumed to be absorbed and is as low as 0.0005% of the bird's total GLDA consumption during the trial period. Taking into account the low toxicity profile of GLDA (Braun et al., 2012), these values are considered as very low and do not pose any safety risk. The higher accumulation of GLDA in kidney compared to liver and muscle reflects the renal pattern of excretion, indicating that very likely the small fraction of GLDA that is absorbed is actively excreted by the animal, as shown with EDTA (Foreman et al., 1953; Heimbach et al., 2000).

The limited absorption of GLDA indicates that the role of GLDA affecting Zn availability takes place within the gastrointestinal tract of the animal, by sustaining solubility during digestive processes as described for other chelating agents (Krezel and Maret, 2016). The complex of GLDA with a mineral is not expected to be absorbed; it only mediates in the availability of the mineral ion for absorption, relying on active and controlled uptake. Chelation of GLDA is expected to support trace mineral homeostasis of the animal, as active and controlled uptake is downregulated when sufficient minerals are present (Windisch, 2002; Richards et al., 2010).

The lowest no-observed-adverse-effect-level found in animal studies with GLDA is 300 mg/kg body weight per day (ECHA, 2010). When an overall uncertainty factor of 100 is applied, one can derive an acceptable daily intake (**ADI**) of 3 mg GLDA/kg bodyweight. The exposure to GLDA of consumers of edible chicken tissue derived from birds receiving GLDA was calculated using the theoretical daily human consumption of tissues from birds of the standard food basket, that is, 150 g of breast meat, 50 g of liver and 5 g of kidney and the residues of GLDA (mean value plus 3 × SD) detected in edible tissue at 10000 mg/kg GLDA inclusion (Additives and Feed, 2012; Sui et al., 2017). This results in a theoretical intake of GLDA of 0.004 mg/kg body weight per day in a 60‐kg adult which is only 0.13% of the ADI (3 mg GLDA/kg bodyweight). The consumption of edible tissue from chickens fed GLDA at 100 times the lowest recommended level of 100 mg GLDA/kg complete feed would result in an exposure to GLDA many fold lower than the ADI and therefore, in daily practice at much lower dosing levels, it is not likely to pose a safety concern for the consumer.

In conclusion the data indicate that dietary GLDA inclusion up to 3,000 mg/kg did not result in any adverse effects on performance, hematology and plasma chemistry. The inclusion of GLDA at 10,000 mg/kg resulted in a reduction in performance, most likely due to compromised barrier function, potentially explained by a combination of direct effects of the chelator and the high sodium load present in the salt form administered. Necropsy results however did not show any pathological changes or indications of severe adverse health effects in any of the inclusion levels. Moreover, GLDA residue levels showed low levels (<0.01%) in breast tissue even when dosed at 100 times the (lowest) recommended dose, for example, 10,000 mg/kg. The residue levels in liver and kidney indicated that the small fraction of GLDA that is absorbed is actively excreted. The residue results indicate that GLDA supports the active transcellular transport system of Zn, thereby supporting Zn homeostasis. The present study reveals a high level of tolerance and safety for GLDA in the broilers and demonstrates no consumer risk of inadvertently increased GLDA intake through consumption of chicken meat or liver from poultry supplemented at a dose of up to 3,000 mg/kg of GLDA/kg feed.

## DISCLOSURES

Some of the authors are employed by Trouw Nutrition, a company that has commercial interests in mineral nutrition of food producing animals. Trouw Nutrition R&D adheres to the principles of the European Code of Conduct for Research Integrity (Drenth, 2012).
